# The Impact of Glucose Oxidase Immobilization on Dendritic Gold Nanostructures on the Performance of Glucose Biosensors

**DOI:** 10.3390/bios12050320

**Published:** 2022-05-10

**Authors:** Laura Sakalauskiene, Anton Popov, Asta Kausaite-Minkstimiene, Arunas Ramanavicius, Almira Ramanaviciene

**Affiliations:** 1 NanoTechnas—Center of Nanotechnology and Materials Science, Institute of Chemistry, Faculty of Chemistry and Geosciences, Vilnius University, Naugarduko Str. 24, LT-03225 Vilnius, Lithuania; laura.sakalauskiene@chgf.vu.lt (L.S.); anton.popov@chgf.vu.lt (A.P.); asta.kausaite@chf.vu.lt (A.K.-M.); arunas.ramanavicius@chf.vu.lt (A.R.); 2 Department of Immunology, State Research Institute Centre for Innovative Medicine, Santariskiu Str. 5, LT-08406 Vilnius, Lithuania

**Keywords:** glucose oxidase, dendritic gold nanostructures, enzyme immobilization methods, electrochemical glucose biosensor

## Abstract

In recent years, many efforts have been made to develop rapid, sensitive and user-friendly glucose biosensors for monitoring blood glucose concentration in patients. In this study, the electrochemical glucose biosensors based on graphite rod (GR) electrode electrochemically modified with dendritic gold nanostructures (DGNs) and glucose oxidase (GOx) were developed. Phenazine methosulfate was used as a soluble redox mediator. Three GOx immobilization methods: adsorption on DGNs and cross-linking with glutaraldehyde (GA) vapour (GA-GOx/DGNs/GR), covalent immobilization on DGNs modified with 11-mercaptoundecanoic acid self-assembled monolayer (SAM) (GOx-SAM/DGNs/GR) and covalent immobilization on SAM with additional cross-linking with GA vapour (GA-GOx-SAM/DGNs/GR), were used. It was determined that GA significantly improved the stability of the enzyme layer. The difference of maximal current generated during the enzymatic reaction (Δ*I_max_*) equal to 272.06 ± 8.69 µA was obtained using a biosensor based on GA-GOx/DGNs/GR electrodes. However, the highest Δ*I_max_* equal to 384.20 ± 16.06 µA was obtained using GA-GOx-SAM/DGNs/GR electrode. Δ*I_max_* for biosensors based on the GA-GOx-SAM/DGNs/GR electrode was 1.41 times higher than for the GA-GOx/DGNs/GR, whereas the linear dynamic range from 0.1 to 10 mM was the same using all three GOx immobilization methods. The limit of detection using GA-GOx-SAM/DGNs/GR and GA-GOx/DGNs/GR electrodes was 0.019 and 0.022 mM, respectively. The ability to detect glucose in the serum by developed biosensors was evaluated.

## 1. Introduction

An important task of modern medicine is the comprehensive and accurate diagnosis of diseases and monitoring concentration of the biologically important substances in body fluids. Today, diabetes mellitus, a chronic metabolic disease commonly known as diabetes, has become one of the greatest health problems in the world [1]. In a healthy person, the fasting glucose level in the blood ranges from 3.9 to 5.6 mM [2], whereas glucose levels in diabetic patients exceed these levels. Blood glucose concentration below or above the normal range may be an indicator of a medical condition. Thus, sensitive, selective, stable, fast, and user-friendly analytical tools are required for the diagnosis and treatment of diabetic patients.

Various analytical methods, mainly optical and electrochemical, are used to determine the glucose content in biological fluids [3,4]. Among these methods, electrochemical enzymatic biosensors based on glucose oxidase (GOx) are the most widely used due to their simplicity, high sensitivity and selectivity, proper linear range, and low limit of detection (LOD) [5,6]. However, the performance of biosensors can be improved by the depositing of nanostructures on the electrode surface [7] and by the selecting the most favourable GOx immobilization method [8]. Direct deposition of gold nanostructures (GNs) on the electrode surface using constant potential amperometry (CPA), pulse amperometry, differential pulse voltammetry, and square-wave voltammetry is a simple and highly efficient method ensuring the formation of different shape and size nanostructures [9,10,11]. GNs are often used in the development of different types of biosensors [12,13]. Additionally, GNs are becoming popular as electrode materials for biofuel cells. GNs-modified electrodes have a large surface area that allows higher enzyme loading, better orientation and more efficient electrical contact [14], excellent chemical stability and resistance to surface poisoning during electrochemical processes, high conductivity, catalytic efficiency, and biocompatibility [15]. Due to these properties, GNs-modified electrodes enhance the electrocatalytic activity of enzymes [16], sometimes provide direct electron transfer [17], and improve the performance of biofuel cells [18,19].

Among different shaped GNs, the dendritic gold nanostructures (DGNs) have been greatly successful due to their superhydrophobicity [20], unique size, and shape-dependent properties, such as enhanced SERS sensitivity [21,22], electrocatalytic activity [23], and photoluminescence emission [24]. The most convenient way to synthesize DGNs on a graphite electrode in one step without any template, seeds, surfactants, or stabilizers is CPA. It was experimentally determined that the optimal electrodeposition potential is −0.4 V for 400 s at a 6.0 mM concentration of HAuCl_4_ [11].

The selection of the best enzyme immobilization method on the electrode is of high importance in the development of well-functioning amperometric glucose biosensors. The most commonly used enzyme immobilization methods are adsorption or covalent attachment, whereas cross-linking of the enzyme on the electrode by specific reagents, covalent immobilization or entrapment in the conducting polymer layer, and encapsulation in nanoparticles or microcapsules are also applied [12,25,26]. Adsorption is the fastest and simplest method, but the enzyme layer is characterized by low stability and the electrodes by low reproducibility. The covalent immobilization of the GOx on self-assembled monolayer (SAM) ensures spatial control over the enzyme [27]. Glutaraldehyde (GA) is a well-known potent cross-linker with reactive aldehyde groups. It can be used for surface activation and covalent enzyme immobilization on different surfaces or as a cross-linker via amino groups after enzyme adsorption on the electrode surface. The use of GA in the development of electrochemical glucose biosensors might be a critical component for the formation of a stabile enzyme layer on the electrode [8]. It has been experimentally confirmed that GA may change the enzyme conformation and activity, depending on its concentration and crosslinking time. For this reason, the milder conditions of crosslinking with GA vapour were elaborated [28].

The aim of this study was to compare different GOx immobilization methods on DGNs in order to develop a stable, selective, and sensitive amperometric glucose biosensor that functions well in real samples. The electrochemical deposition of DGNs on the electrode surface was optimized using the CPA method. Three GOx immobilization methods, namely, simple adsorption on DGNs and cross-linking with GA vapour (first method), covalent immobilization on DGNs modified with 11-mercaptoundecanoic acid SAM (second method), and covalent immobilization on SAM with additional cross-linking with GA vapour (third method) were used and the performance of the developed glucose biosensors was characterized.

## 2. Materials and Methods

### 2.1. Materials

Glucose oxidase (type X-S from Aspergilus niger, 228.3 U mg^−1^ protein) and ascorbic acid C_6_H_8_O_6_ were received from Sigma-Aldrich (St. Louis, MO, USA). Phenazine methosulfate (PMS), uric acid C_5_H_4_N_4_O_3_, and citric acid C_6_H_8_O_6_ were obtained from AppliChem GmbH (Darmstadt, Germany). D-(+)-Glucose, D-(+)-galactose, D-(+)-xylose, D-(+)-mannose, and hydrogen tetrachloroaurate (III) trihydrate (HAuCl_4_·3 H_2_O) were purchased from Carl Roth GmbH&Co (Karlsruhe, Germany). Potassium nitrate (KNO_3_), potassium chloride (KCl), and N-hydroxysuccinimide (NHS) were acquired from MERCK KGaA (Darmstadt, Germany). Sodium acetate trihydrate, 11-Mercaptoundecanoic acid (MUA), methanol, D-(+)-Saccharose and 1-ethyl-3-(3-dimethylaminopropyl)carbodimide hydrochloride (EDC) were obtained from Sigma-Aldrich Chemie GmbH (Steinheim, Germany). Sodium citrate was received from Penta (Praha, Czech Republic) and 25% glutaraldehyde (GA) from Fluka Chemie GmbH (Buchs, Switzerland). Graphite rods (GR) (diameter 3.0 mm, purity—99.999%) were purchased from Sigma-Aldrich (St. Louis, MO, USA) and alpha alumina powder (grain diameter 0.3 μm, Type N) from Electron Microscopy Sciences (Hatfield, MA, USA). The sodium acetate (SA) buffered solution (0.05 M CH_3_COONa) with 0.1 M KCl was prepared by mixing sodium acetate trihydrate and potassium chloride. A GOx solution of 40 mg mL^−1^ concentration was prepared in SA (pH 6.5). Before investigations, the glucose solution was allowed to stay overnight for the formation of α-β optical isomers at equilibrium. All other chemicals used in the present study were either analytically pure or of higher quality. All solutions were prepared using deionized water purified with the water purification system Millipore S.A. (Molsheim, France).

### 2.2. Instrumentation

The electrochemical deposition of DGNs and electrochemical measurements were performed using computerized potentiostat/galvanostat Autolab PGSTAT30 (EcoChemie, The Netherlands) driven by NOVA1.9 software. The electrochemical cell was composed of a GR electrode (unmodified and premodified) as a working electrode, Ag/AgCl/KCl_3M_ as a reference electrode, and Pt wire as a counter electrode. Morphological studies of GR electrodes modified by electrochemically deposited DGNs were characterized by a high- resolution field emission scanning electron microscope SU-70 (Hitachi, Japan) (FE-SEM). Energy-dispersive X-ray spectroscopy (EDS) using scanning electron microscope Hitachi TM3000 (Hitachi, Japan) at 15 kV accelerating voltage was applied for determination of the chemical compositions of prepared electrodes.

### 2.3. Pretreatment of the Working Electrode and Electrochemical Deposition of DGNs

The graphite rods were broken and mechanically polished with fine (P120), very fine (P320), and finally, with ultra-fine grit (P2000) sandpaper followed by GR electrodes washing with ethanol and UHQ water and drying at room temperature. Then, the side surface of GR electrodes was isolated with a silicone tube in order to avoid its contact with solution in the electrochemical cell. The working surface area of GR electrodes is 0.071 cm^2^. The GR electrode was immersed into an electrochemical cell filled with an aqueous solution consisting of 10 mM HAuCl_4_ and 0.1 M KNO_3,_ and an electrochemical deposition of DGNs was performed at a constant −0.2 V potential vs. Ag/AgCl/KCl_3M_ for 200 or 400 s. After DGNs synthesis, the electrode was rinsed with deionized water and dried at room temperature. The surface of GR electrodes modified by electrochemically deposited DGNs (DGNs/GR) was characterized by a FE-SEM.

To determine the electroactive surface area of electrodeposited DGNs, cyclic voltammogram was performed in 0.5 M H_2_SO_4_ electrolyte solution. Cyclic voltammogram was recorded in the potential range from 0 to +1.4 V vs. Ag/AgCl/KCl_3M_ using the scan rate of 0.1 V/s. The integrated peak of gold oxide reduction and the conversion factor of 386 μC cm^−2^ [29] were used for calculation of the electroactive surface area of gold [30].

### 2.4. Immobilization of GOx on a DGNs/GR Electrode

Three different GOx immobilization methods on the GR electrode premodified with electrochemically deposited DGNs were used and compared in this study. Namely, GOx was immobilized by simple adsorption on DGNs and cross-linked with the GA vapour (GA-GOx-DGNs/GR) (first method), covalently on DGNs modified with 11-MUA SAM (GOx-SAM/DGNs/GR) (second method), and covalently on 11-MUA SAM with additional cross-linking with the GA vapour (GA-GOx-SAM/DGNs/GR (third method). In case of the first method, the DGNs/GR electrode was immersed in a tube filled with 40 mg mL^−1^ GOx solution for 30 min and dried at room temperature (+20 ± 2 °C). Then, the working electrode was kept for 15 min in a closed vessel over a 25% solution of GA at room temperature. The selection optimal time for the cross-linking of GOx with the GA vapour is presented in Figure S1 (in Supplementary Materials). In the second method, the DGNs/GR electrode was immersed in 1 mM 11-MUR solution in methanol and stored for 2 h. After rinsing the working electrode with methanol and deionized water, the carboxyl groups of 11-MUA SAM were activated by a mixture of 50 mM EDC and 200 mM NHS aqueous solutions (ratio 1:1) for 15 min. The electrode was then rinsed with SA and immersed in a tube filled with 40 mg mL^−1^ GOx solution for 30 min to covalently immobilize GOx. In the third method, GOx was immobilized the same way as it was done in second method, but additional cross-linking with the GA vapour was applied. Thus, the GOx-SAM/DGNs/GR electrode was kept for 15 min in a closed vessel over a 25% solution of GA at room temperature. Before all electrochemical measurements, GOx-modified electrodes were washed with distilled water to remove non cross-linked enzymes. All working electrodes were stored in a closed vessel over SA buffer at +4 °C before use. Prior to electrochemical measurements, the electrodes were washed with UHQ water.

### 2.5. Electrochemical Measurement

**Scheme 1:**
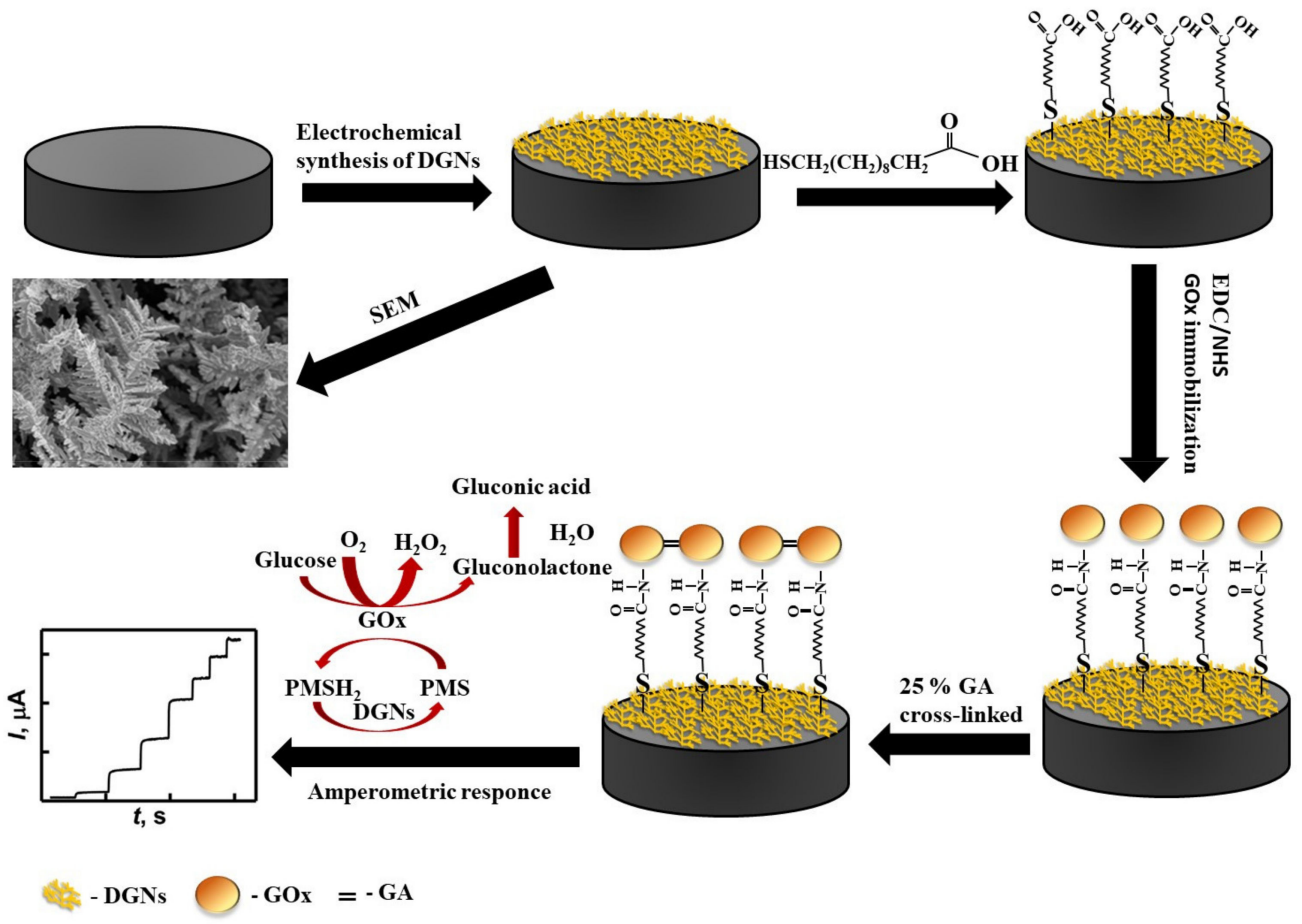
Schematic representation of the modification of the GR electrode by DGNs, immobilization of GOx using the third method, and electrochemical determination of glucose by the developed biosensor.

The amperometric signals of biosensors using GR electrodes premodified with DGNs and GOx by three immobilization methods were evaluated in 0.05 M SA buffer with 0.1 M KCl (pH 6.0) and PMS. Firstly, a steady baseline current was reached, and then different concentrations of glucose were added. During the enzymatic oxidation of β-D-glucose, two molecules of flavin adenine dinucleotide (FAD) tightly bound to each GOx subunit are reduced. The artificial redox mediator PMS present in the electrochemical cell is involved in the reoxidation of the GOx redox center, and the resulting PMSH_2_ (the reduced form of PMS) is reoxidized on the surface of the working electrode (Scheme 1). Electrons from the PMSH_2_ are transferred to the positively charged electrode in two ways: directly to the GR electrode and through GNs [31]. Currents at +0.3 V potential vs. Ag/AgCl/KCl_3M_ were recorded at glucose concentrations in the range from 0.1 to 100 mM. The current response under certain concentrations of glucose (Δ*I*) was evaluated as the difference of registered steady anodic currents after the addition of glucose and baseline. All investigations were carried out at +20 ± 2 °C temperature in a 5 mL volume electrochemical cell stirring solution with a magnetic stirrer (120 rpm). The results of all electrochemical measurements are reported as the mean values of three independent experiments.

### 2.6. Calculations

Amperometric signals showed hyperbolic dependence on glucose concentrations and it was in good agreement with the Michaelis–Menten kinetics. The difference of maximal currents generated during the enzymatic reaction (Δ*I*_max_) and the apparent Michaelis constant (*K*_M(app.)_) were, correspondingly, the parameters *a* and *b* of the hyperbolic function *y = ax/(b+x)*, which was used for the approximation of the results. Parameters of the enzyme-catalyzed reaction were calculated using Sigma-Plot software (version 11.00). The calibration curve parameters, such as correlation coefficient (*R*) and determination coefficient (*R*^2^), were evaluated. The LOD was calculated as the lowest concentration of the analyte, which gives an analytical signal greater than the background value plus 3σ.

## 3. Results

### 3.1. Optimization of Electrochemical Deposition of DGNs

DGNs can be synthetized from different concentrations of HAuCl_4_ solutions, using various methods and different durations of synthesis. In our previous study [11], DGNs were synthesized from a 6.0 mM solution of HAuCl_4_ at a constant −0.4 V potential vs. Ag/AgCl/KCl_3M_ reference electrode for 400 s.

**Figure 1:**
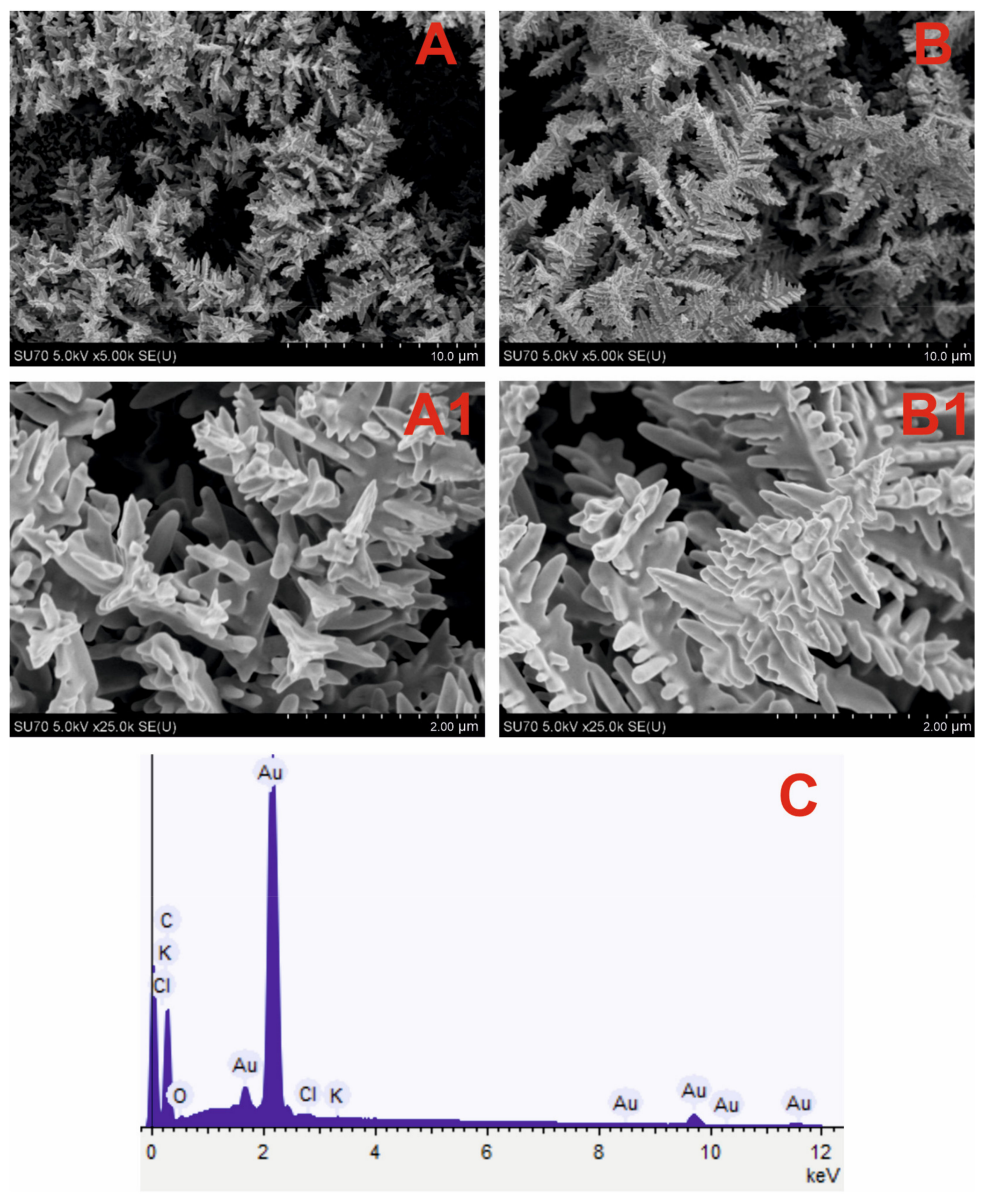
SEM images of the DGNs electrochemically deposited on a GR electrode at a constant −0.2 V potential for 200 s (**A**,**A1**) and 400 s (**B**,**B1**) from a 10 mM solution of HAuCl_4_ containing 0.1 M KNO_3_ (pictures (**A1**,**B1**) are magnified images of (**A**,**B**)) and EDS spectrum (**C**) of dendritic gold nanostructures presented in (**A**) SEM image.

GR electrode premodified with DGNs synthetized at these conditions was applied for the development of the glucose biosensor. In this study, we decided to test the influence of a 10 mM concentration of HAuCl_4_ on the morphology of DGNs synthesized at a constant −0.2 V potential for 200 s and 400 s. FE-SEM images (Figure 1A,B) confirmed that at both conditions DGNs were successfully electrochemically deposited on the GR electrode. The longer, more ordered DGNs were formed at the synthesis duration of 400 s (Figure 1B,B1), whereas shorter, sharper, and more branched DGNs were formed after 200 s (Figure 1A,A1).

The chemical composition of premodified electrodes was determined using EDS analysis, wherein similar results were obtained in both cases. The EDS spectrum of the DGNs electrodeposited for 200 s on the GR electrode (Figure 1C) indicates the predominance of Au and C elements. Moreover, other characteristic peaks correlated with Cl, K, and O elements were obtained and could be explained by the incomplete washing of electrodes from the substances used during the synthesis.

**Figure 2:**
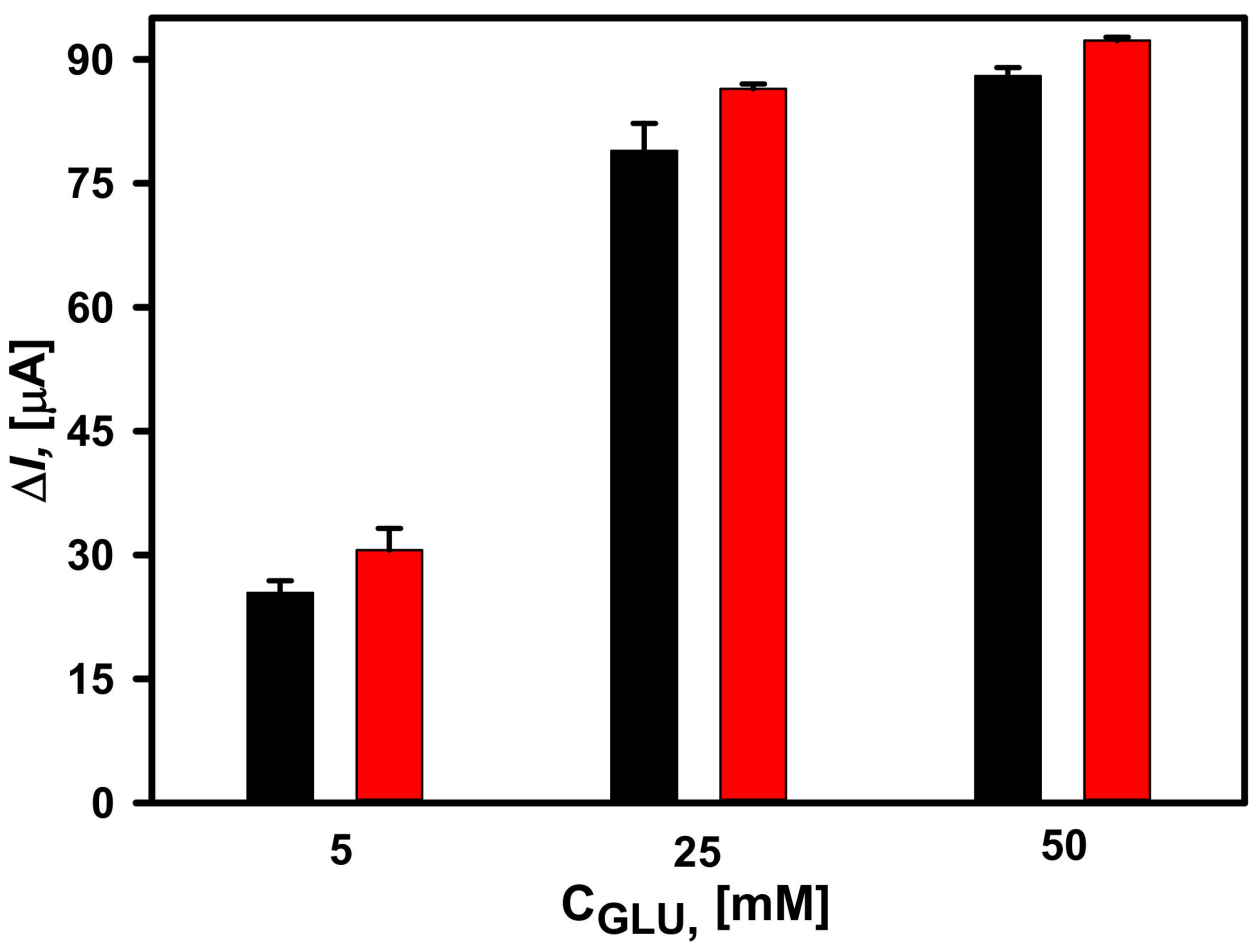
Dependence of the current response of glucose biosensors based on electrochemically deposited DGNs from a 10 mM HAuCl_4_ solution with 0.1 M KNO_3_ at a constant −0.2 V potential for 200 s (red column) and 400 s (black column). The amperometric response at +0.3 V was registered in 0.05 M SA buffer with 0.1 M KCl (pH 6.0) and 2.0 mM PMS.

The difference in morphology and chemical composition was not so obvious, thus the effect of DGNs synthesis on the performance of the glucose biosensor was tested electrochemically. The third GOx immobilization method (GA-GOx-SAM/DGNs/GR) was selected for this study. It is obviously seen that amperometric signals obtained at glucose concentrations of 5, 25 and 50 mM were similar for both DGNs electrodeposition times (Figure 2). However, for all concentrations measured, a higher current response was registered when shorter electrodeposition duration was used. This effect was especially noticeable at lower glucose concentrations. For instance, at 5 mM glucose, Δ*I* decreased 1.2 times when DGNs were deposited for 400 s. Thus, for the further studies, it was decided to use a GR electrode with DGNs electrochemically deposited at −0.2 V potential for 200 s.

**Figure 3:**
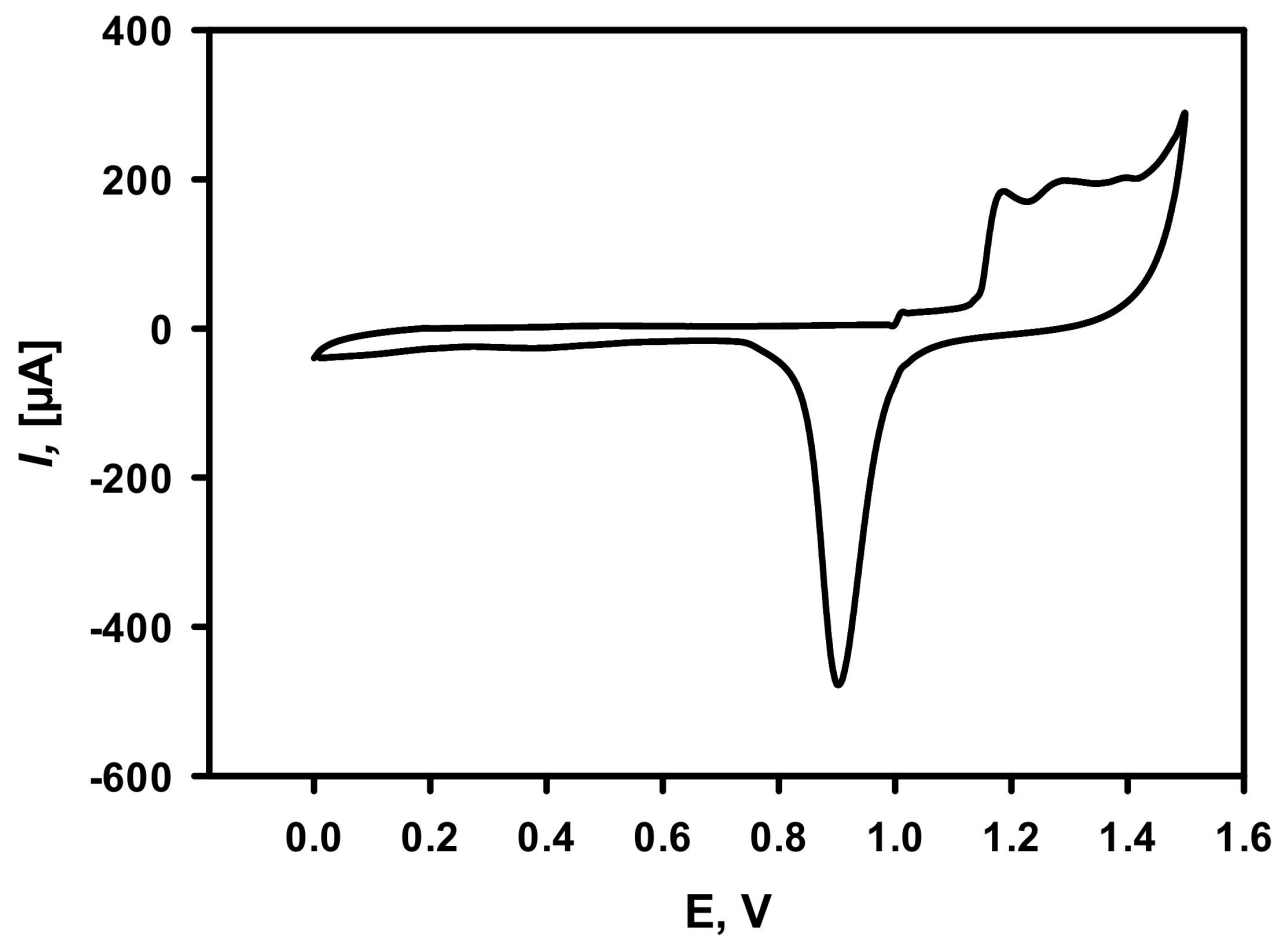
Cycling voltammogram of DGNs deposited on the GR electrode recorded in 0.5 M H_2_SO_4_. The scan rate was 0.1 V/s. DGNs electrodeposition was performed at a constant −0.2 V potential for 200 s from a 10 mM solution of HAuCl_4_ containing 0.1 M KNO_3_.

Additionally, cyclic voltammetry measurement (Figure 3) in 0.5 M H_2_SO_4_ was performed for the calculation of the electroactive surface area of electrodeposited DGNs. It is obvious that electrochemical gold oxidation and further reduction take place. The gold electroactive surface area was calculated to be equal to 1.03 ± 0.15 cm^2^.

### 3.2. Comparison of Different GOx Immobilization Methods on the Performance of Glucose Biosensors

**Figure 4:**
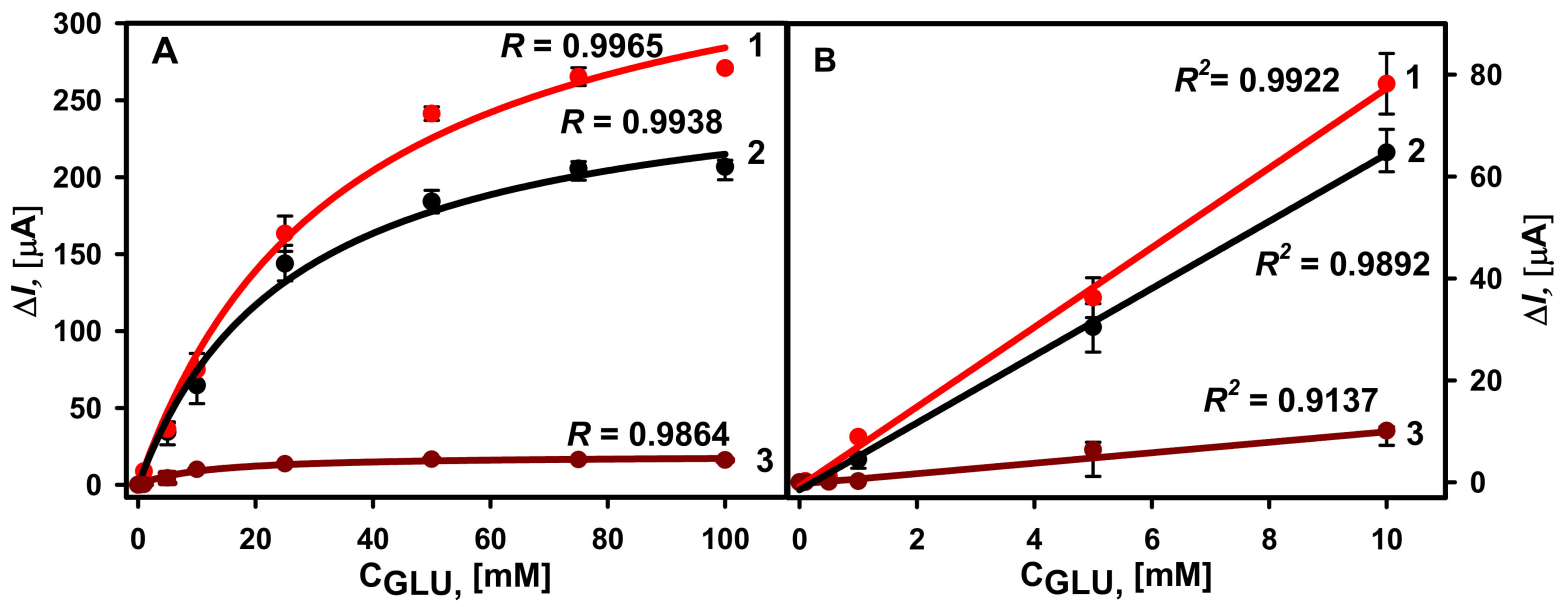
(**A**) Calibration plots of glucose biosensors based on GA-GOx-SAM/DGNs/GR (curve 1, third GOx immobilization method, red dots ●), GA-GOx/DGNs/GR (curve 2, first GOx immobilization method, black dots ●), and GOx-SAM/DGNs/GR electrodes (curve 3, second GOx immobilization method, brown dots ●). (**B**) The linear dynamic ranges of the developed biosensors. The amperometric response at +0.3 V was registered in 0.05 M SA buffer with 0.1 M KCl (pH 6.0) in the presence of 6.0 mM PMS.

Following the electrochemical synthesis of DGNs, GOx was immobilized on the electrode surface in three different ways, as described in Section 2.4. Amperometric response to different glucose concentrations was registered in 0.05 M SA buffer (pH 6.0) with 6.0 mM PMS. The selection of optimal PMS concentration is presented in Figure S2. During the enzymatic reaction in the presence of redox mediator, electrons are transferred toward the working electrode surface and the steady-state current is registered at +0.3 V potential vs. Ag/AgCl/KCl_3M_. The amperograms for all three types of electrodes are presented in Figures S3–S5. The hyperbolic relationship between registered currents and glucose concentrations in the range from 0.1 to 100 mM was obtained for all calibration curves (Figure 4A). The lowest Δ*I_max_* equal to 19.21 ± 0.75 µA (*R* = 0.9864) was obtained using the second GOx immobilization method, in which GOx was covalently immobilized on SAM/DGNs/GR without additional crosslinking (GOx-SAM/DGNs/GR). The 14.2 times higher Δ*I_max_* equal to 272.06 ± 8.69 µA (*R* = 0.9938) was obtained using the first GOx immobilization method when GOx was adsorbed on DGNs and cross-linked with GA vapour (GA-GOx/DGNs/GR). The *K*_M(app.)_ was equal to 26.56 ± 2.42 mM, and 20 times higher Δ*I_max_* equal to 384.20 ± 16.06 µA (*R* = 0.9965) was obtained using the third method, in which covalent GOx immobilization and a further 15 min of lasting cross-linking with 25% vapour of GA were used. In this case, the *K*_M(app.)_ was a little bit higher and equal to 35.23 ± 3.76 mM. The highest current response and Δ*I_max_* were obtained after GOx covalent immobilization on the 11-MUA SAM with additional crosslinking by the GA vapour (GA-GOx-SAM/DGNs/GR). Δ*I_max_* for the glucose biosensor based on the GA-GOx-SAM/DGNs/GR electrode was 1.41 times higher than for the GA-GOx/DGNs/GR electrode, whereas the linear dynamic range (LDR) from 0.1 to 10 mM was the same using all three GOx immobilization methods (Figure 4B). The LOD was lower for the biosensor based on the GA-GOx-SAM/DGNs/GR electrode at 0.019 mM, whereas for the GA-GOx/DGNs/GR electrode LOD it was 0.022 mM.

Summarizing the results obtained, we can state that the performance of glucose biosensor is better using GR electrode premodified with DGNs and GOx by the third method. The requirement of additional cross-linking of GOx covalently immobilized on the SAM was also observed by other authors. The 0.05% *v*/*v* GA was additionally used for cross-linking covalently immobilized GOx on the aldehyde carrier at room temperature for about 2 h. This carrier was obtained after NH_2_-terminated monolayer interaction with GA. Such requirements for GOx immobilization might be explained by a large size of GOx (ca. 160 kDa) in comparison to smaller enzymes, such as horseradish peroxidase, and random distribution of amino groups on the surface of enzyme [32]. The comparison of GOx immobilization methods on the performance of second generation glucose biosensors based on application of different gold nanostructures is presented in Table 1. The developed glucose biosensors with DGNs were characterized by low LOD and appropriate linear range for the glucose detection.

### 3.3. Study of Analytical Signal Repeatability

**Figure 5:**
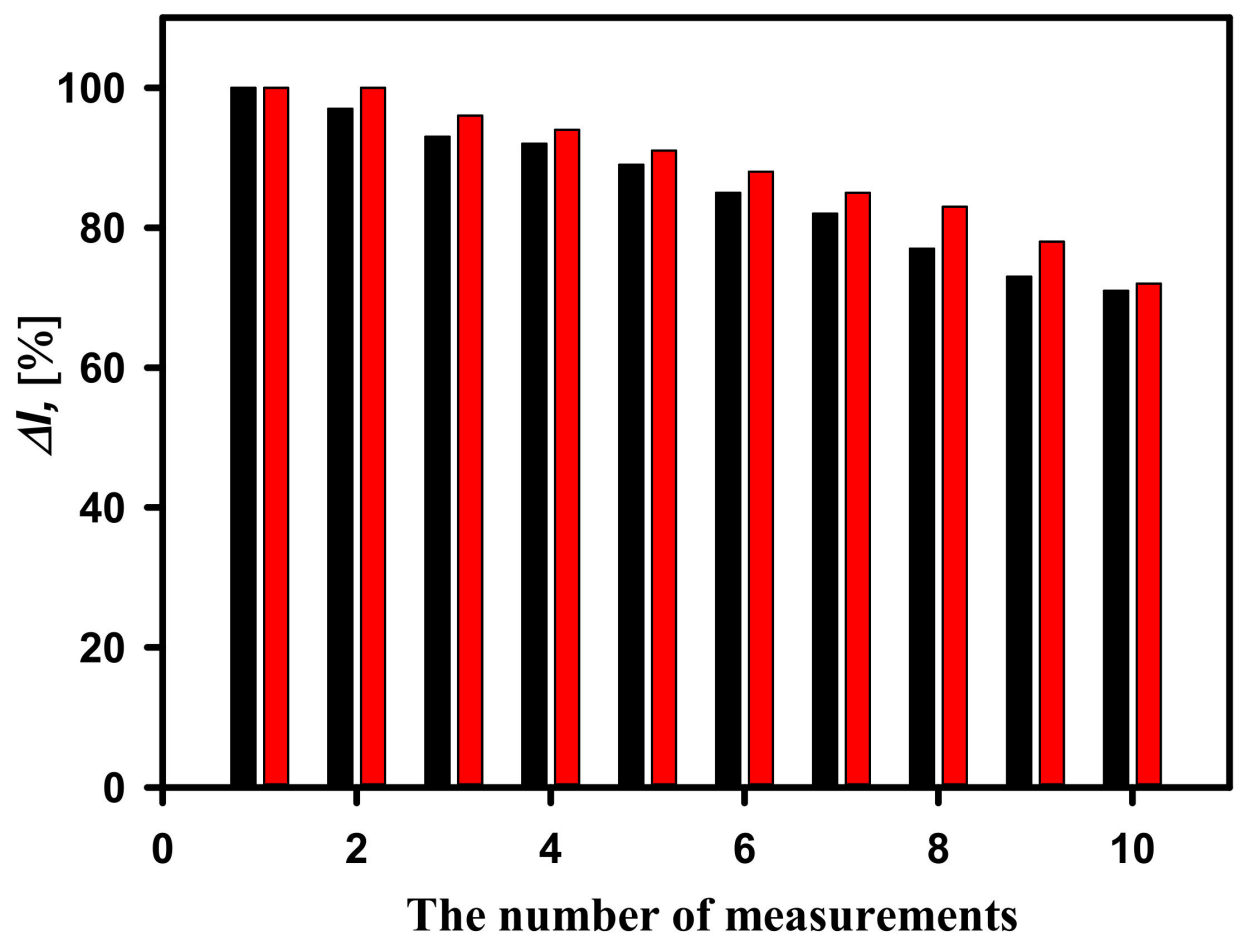
Current responses of the developed biosensors based on GA-GOx-SAM/DGNs/GR electrode (red columns) and GA-GOx/DGNs/GR electrodes (black columns) during repeated analyte detection. The current response was recorded at 25 mM of glucose concentration.

Repeatability is one of the important characteristics of biosensors showing the ability to generate similar results during multiple measurements of the same quantity of analyte. Despite the fact that the highest current response was registered by the GA-GOx-SAM/DGNs/GR electrode, the GA-GOx/DGNs/GR electrode was also evaluated. In the repeatability study, the current response was recorded by detecting 25 mM glucose 10 times by adding glucose to the electrochemical cell. As shown in Figure 5, better repeatability was observed using a biosensor based on the GA-GOx-SAM/DGNs/GR electrode. The current response was the same after two measurements, whereas it decreased by 4% after the third measurement, indicating good repeatability, and decreased by 9% after the fifth measurement. A similar but a slightly worse repeatability was obtained by the biosensor based on the GA-GOx/DGNs/GR electrode. The current response after the second measurement decreased by 3%, whereas after the third—7%, and after the fifth measurement, it decreased by 12%. A total of 91% of the initial current response was retained after five measurements using the GA-GOx-SAM/DGNs/GR electrode, whereas 88% was retained using the GA-GOx/DGNs/GR electrode. Subsequent measurements show a slight decrease in the analytical signal for both electrodes. We can conclude that the better repeatability was observed using the biosensor based on the GA-GOx-SAM/DGNs/GR electrode. This effect might be due to a more favorable arrangement of GOx molecules after covalent immobilization on the electrode premodified with 11-MUA SAM and a lower loss of enzyme due to additional crosslinking with GA.

### 3.4. Detection of Glucose in Human Serum Sample

Serum glucose levels for people with diabetes is much wider than in healthy people—from 2 to 30 mM. Thus, the suitability of GA-GOx-SAM/DGNs/GR and GA-GOx/DGNs/GR electrodes was tested for glucose determination in diluted human serum. Before the use, serum was taken from the freezer, thawed, and filtered. Then, it was diluted five times with 0.05 M SA buffer (pH 6.0), containing 0.1 M KCl.

**Figure 6:**
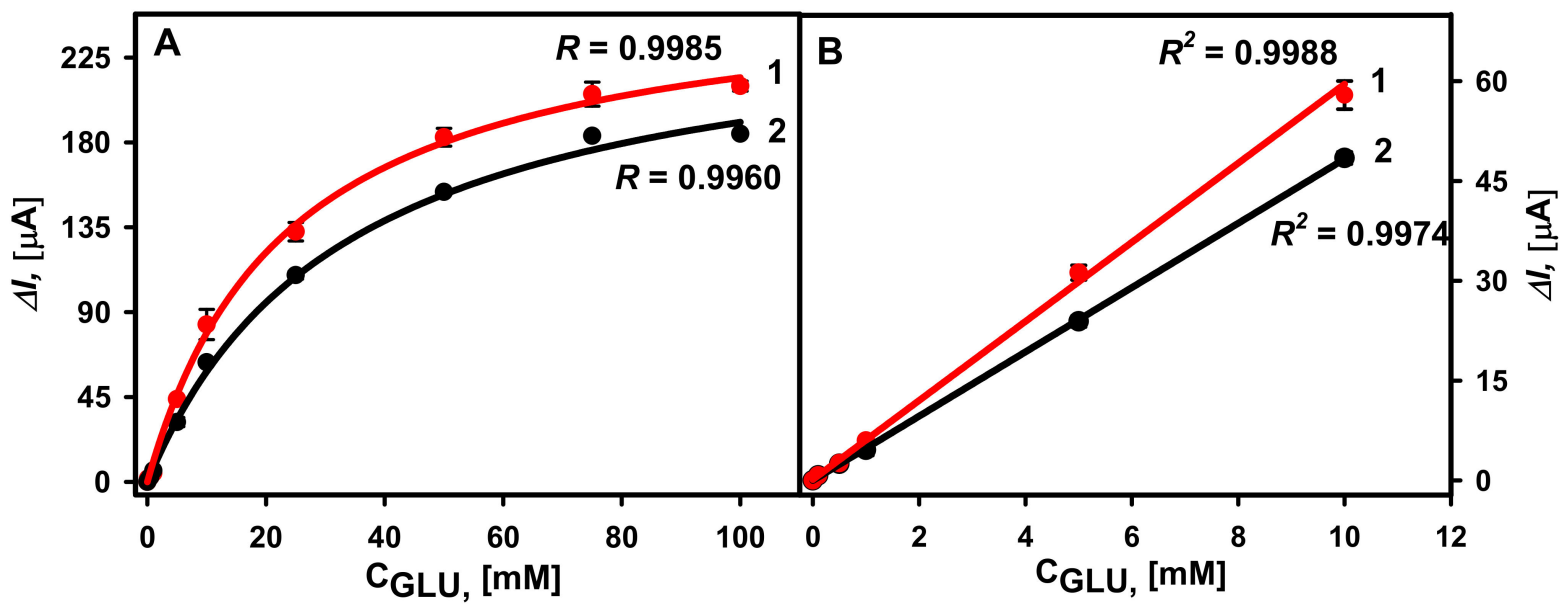
(**A**) Calibration plots of glucose biosensors based on GA-GOx-SAM/DGNs/GR (curve 1, red dots ●) and GA-GOx/DGNs/GR (curve 2, black dots ●) electrodes in serum. (**B**) The linear glucose detection range. The amperometric response at +0.3 V was registered in 0.05 M SA buffer with 0.1 M KCl (pH 6.0) diluted serum in the presence of 6.0 mM PMS.

The hyperbolic relationships between registered currents in the diluted serum and added glucose concentrations in the range from 0.1 to 100 mM were obtained for the two types of electrodes (Figure 6A). It is obvious that a higher current response was registered using the GA-GOx-SAM/DGNs/GR electrode, where the calculated Δ*I_max_* was equal to 312.58 ± 18.34 µA (*R* = 0.9985), whereas when using the GA-GOx/DGNs/GR electrode, it was 244.12 ± 8.79 µA (*R* = 0.9960). Consequently, the *K*_M(app.)_ were 40.42 ± 5.72 mM and 25.22 ± 2.64 mM, respectively. Despite the difference in the value *of K_M(app.)_*, the LDRs (Figure 6B) were the same and no differences depending on the GOx immobilization method were observed. In both cases, quite wide LDRs from 0.1 to 10.0 mM of glucose were observed. For comparison, the LDRs for glucose biosensors based on gold nanoparticles depend on the size of nanoparticles, the redox mediator used, the selected electrochemical method, the enzyme immobilization method, and many other parameters, but usually are not wide, up to 5–10 mM of glucose. However, the LDR can be extended, covering the electrode surface with polymers. For instance, the linear glucose detection range was extended from 9.96 mM up to 19.9 mM after 21 h lasting synthesis of a polypyrrole layer on the GR electrode electrochemically modified with gold nanoparticles and adsorbed GOx with additional cross-linking by the GA vapour [37].

**Figure 7:**
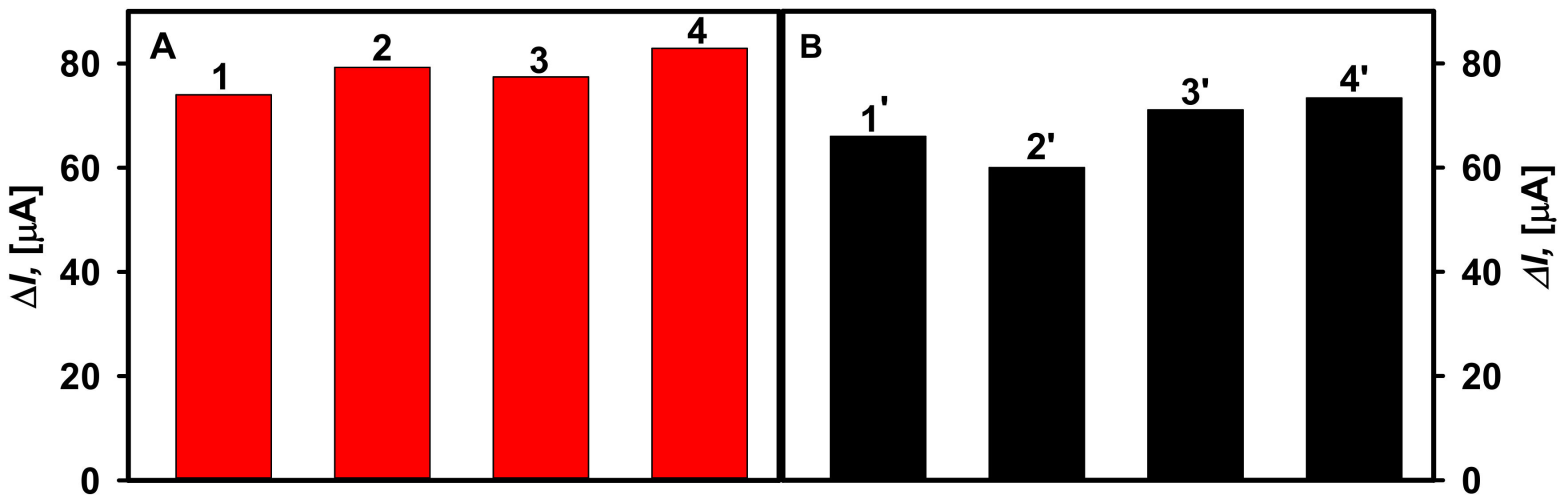
The effect of interfering substances on the current response of the glucose biosensors developed based on GA-GOx-SAM/DGNs/GR (**A**) and GA-GOx/DGNs/GR (**B**) electrodes. Diagrams of the registered current response are presented after addition of: 10 mM solution of glucose (column 1, 1′), 10 mM solution of glucose with 0.1 mM uric acid (column 2, 2′), 10 mM solution of glucose with 0.01 mM ascorbic acid (column 3, 3′), and 10 mM solution of glucose with 0.05 mM ascorbic acid (column 4, 4′).

The selectivity of the developed biosensors was tested after addition of 25.0 mM of galactose, fructose, xylose, and mannose. These substrates had no effect on the registered current responses. Only the addition of 25.0 mM concentration of glucose gives a measurable, current response. Furthermore, the influence of ascorbic and uric acids as possible interfering substances on the determination of glucose was determined (Figure 7). The presence of 0.01 and 0.05 mM ascorbic acid in the solution of 10.0 mM glucose was found to increase the current response by 7.72% and 11.2% using the GA-GOx/DGNs/GR electrode, and by 4.63 and 10.8% using the GA-GOx-SAM/DGNs/GR electrode. In the presence of 0.01 mM of uric acid, the current response increased by 9.09% using the GA-GOx/DGNs/GR electrode, whereas signals registered by GA-GOx-SAM/DGNs/GR decreased by 7.02%. 

A little impact of tested electrochemically active compounds on the registered current response using electrodes modified with GOx according different protocols was observed; however, in most cases the signal difference was less than 10% except in the highest concentrations of ascorbic acid. Additionally, less impact of interfering species was registered by the GA-GOx-SAM/DGNs/GR electrode. Thus, the developed biosensors are suitable for the glucose detection in the serum, and the higher signal to glucose is registered when GOx is covalently immobilized on the 11-MUA SAM and additionally cross-linked with GA. Furthermore, the impact of interfering species on the analytical signals can be reduced by use of electrically conducting polymers formed over the tested electrodes [12,37].

### 3.5. The Stability of the Developed Glucose Biosensors

**Figure 8:**
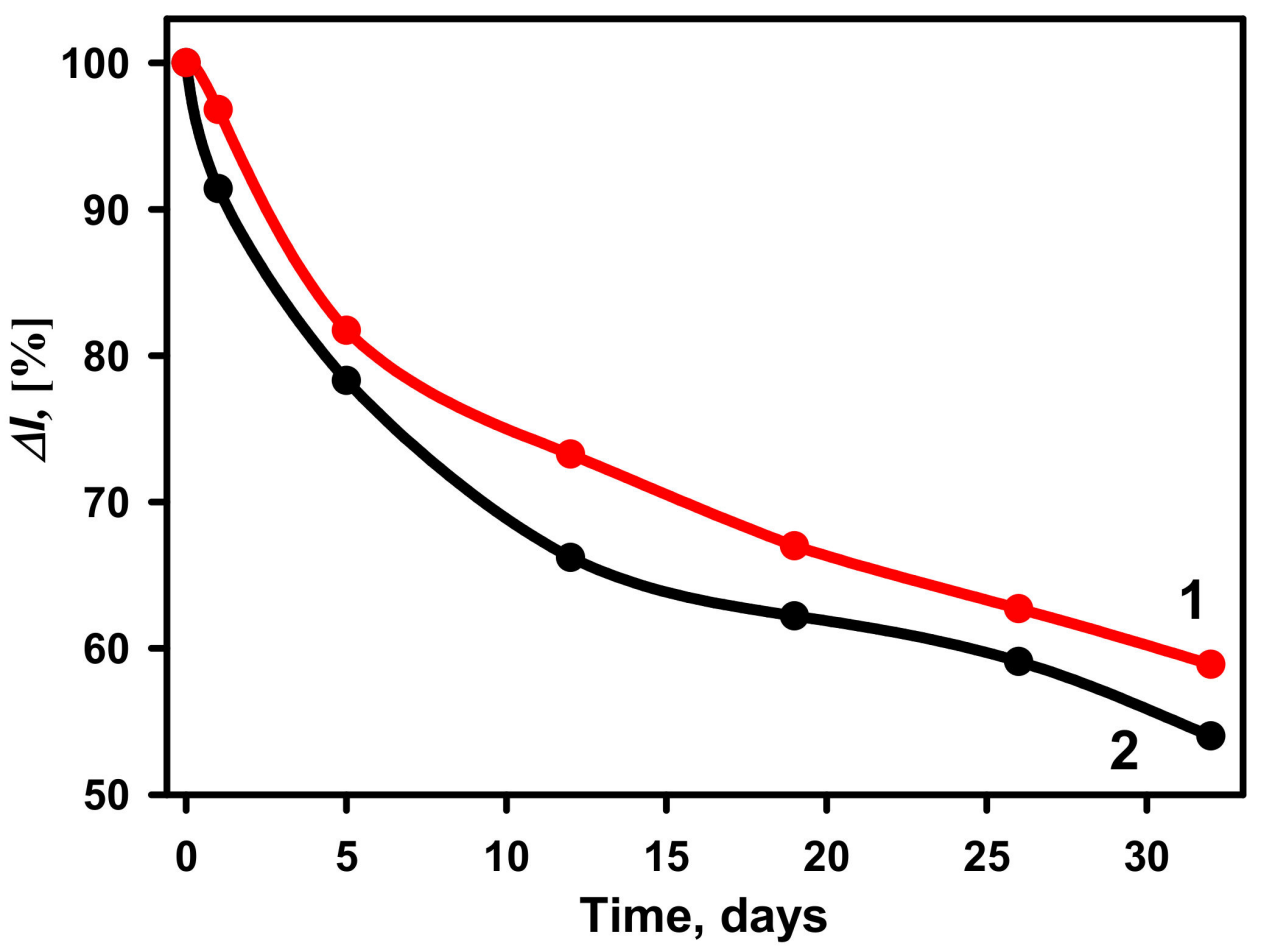
The stability of biosensors based on GA-GOx-SAM/DGNs/GR (curve 1, red dots ●) and GA-GOx/DGNs/GR (curve 2, black dots ●) electrodes. The amperometric response was measured in 0.05 M SA buffer with 0.1 M KCl (pH 6.0) and 6.0 mM PMS.

The stability of glucose biosensors based on GA-GOx-SAM/DGNs/GR and GA-GOx/DGNs/GR electrodes was tested during the 32-day period (Figure 8). The Δ*I%* calculated for both types of biosensors at 50 mM glucose gradually decreased over time. A more rapid decrease in the current response was observed up to 5 days using the GA-GOx-SAM/DGNs/GR electrode, whereas for GA-GOx/DGNs/GR, it takes longer, up to 12 days. After 12 days 73.25% of the initial current response was retained for the GA-GOx-SAM/DGNs/GR electrode and 66.20% for the GA-GOx/DGNa/GR electrode. The GA-GOx-SAM/DGNs/GR electrode was found to have better stability compared to the GA-GOx/DGNs/GR electrode. It might be due to more tightly bound enzyme with proper spatial orientation on more biocompatible surface, so GOx molecules longer are active and stable during the electrochemical measurements and washing setups.

## 4. Conclusions

In this work, the electrochemical deposition of DGNs on a GR electrode at a constant potential in time was optimized. Additionally, it was shown that the selection of GOx immobilization on DGNs method has a great importance on the performance of the developed glucose biosensors in the buffer solution and in the serum. The application of GA to cross-link GOx after covalent immobilization on the SAM significantly improved the sensitivity and stability of the biosensing layer, and repeatability of the current response after multiple detection of glucose with the developed biosensor. Such an immobilization method minimizes the loss of enzyme during continuous amperometric measurements in solution with mixing; however, DGNs are quite fragile and can be damaged or removed from the surface together with enzymes under inappropriate experimental conditions. Furthermore, multilayers of GOx covalently immobilized on gold nanostructures are a very promising direction for improving the analytical parameters of biosensors. In order to simplify the operation of such biosensors, a reagent-less system must be constructed, where the redox mediator will be immobilized on the surface of DGNs/GR electrodes.

## Figures and Tables

**Table 1:** Comparison of glucose oxidase immobilization method on the performance of glucose biosensors with different gold nanostructures.

GOx Immobilization Method	LOD/Sensitivity	LDR	Method/Mediator	Ref.
GOx adsorbed on the dendritic gold nanostructures modified graphite rod electrode and cross-linked with glutaraldehide vapour	0.059 mM	0.1–9.97 mM	Amperometry Phenazine methosulfate	[11]
GOx and 2,5-dihydroxybenzaldehyde cross-linked with glutaraldehyde and adsorbed on nanocomposite consisting of gold nanoparticles/reduced graphene oxide within gelatin deposited on a screen-printed electrode	0.640 mM	1–11 mM	Cyclic voltammetry 2,5-dihydroxybenzaldehyde	[33]
GOx adsorbed on gold nanoparticles (3.5 nm) and redox mediator modified graphite rod electrode and cross-linked with glutaraldehide vapour	0.024 mM 52.1 μA mM^−1^ cm^−2^	0.1–10 mM	Amperometry 1,10-phenanthroline-5,6-dione	[12]
GOx adsorbed on gold nanoparticles electrochemically deposited on graphite rod electrode and cross-linked with glutaraldehide vapour	0.083 mM 101.02 μA mM^−1^ cm^−2^	0.1–10 mM	Amperometry Phenazine methosulfate	[7]
GOx covalent attachment to gold nanoparticles (2.6 nm) monolayer modified gold electrode	0.0082 mmol L^−1^ 8.8 μA mM^−1^ cm^−2^	0.02–5.7 mM	Amperometry Ferrocenemethanol	[34]
GOx covalently immobilised on nanoporous gold modified with self-assembled monolayers (3,3′-dithiodipropionic acid/6-mercaptohexanoic acid/11-mercaptoundecanoic acid) via carbodiimide chemistry	2.187 μA mM^−1^ cm^−2^ 1.564 μA mM^−1^ cm^−2^ 1.160 μA mM^−1^ cm^−2^	0.1–10 mM	Amperometry p-benzoquinone	[35]
Multilayers of GOx covalently immobilised on the gold electrode and gold nanoparticles (12 nm) obtained using cysteamine (6 layers)	0.008 mM 5.72 μA mM^−1^ cm^−2^	0.01–13 mM	Amperometry Ferrocenemethanol	[36]
GOx covalently immobilized on 11-MUA SAM modified DGNs/GR electrode and cross-linked with GA vapour (GA-GOx-SAM/DGNs/GR)	0.019 mM	0.1 to 10 mM	Amperometry Phenazine methosulfate	This work
GOx adsorbed on the DGNs/GR electrode and cross-linked with GA vapour (GA-GOx/DGNs/GR)	0.022 mM	0.1 to 10 mM	Amperometry Phenazine methosulfate	This work

## Data Availability

The data presented in this study are available on request from the first author.

## Supplementary Materials

The following are available online at https://www.mdpi.com/article/10.3390/bios12050320/s1, Figure S1: Calibration plots of glucose biosensors based on GA-GOx-SAM/DGNs/GR electrode without additional cross-linking with 25% glutaraldehyde vapour (curve 1), and after cross-linking for 3 min (curve 2), 10 min (curve 3) and 15 min (curve 4). Figure S2: Selection of the optimal concentration of redox mediator PMS. Experiments were performed using GA-GOx-SAM/DGNs/GR electrode. Figure S3: The amperogram of biosensor based on GOx/SAM/DGNs/GR electrode after addition of glucose. Figure S4: The amperogram of biosensor based on GA-GOx/DGNs/GR electrode after addition of glucose. Figure S5: The amperogram of biosensor based on GA-GOx/SAM/DGNs/GR electrode after addition of glucose.
