# Supplementary material for: The Impact of Glucose Oxidase Immobilization on Dendritic Gold Nanostructures on the Performance of Glucose Biosensors
Source: Biosensors (Basel). 2022 May 10;12(5):320. doi: 10.3390/bios12050320 (PMC9139151; doi:10.3390/bios12050320)
Supplement: Supplementary file 1 [file biosensors-12-00320-s001.zip › biosensors-1685859-supplementary.pdf]

biosensors

MDPI

Supplementary Material

The Impact of Glucose Oxidase Immobilization on Dendritic Gold Nanostructures on the Performance of Glucose Biosensors

Laura Sakalauskiene [1], Anton Popov [1,2], Asta Kausaite-Minkstimiene [1,2], Arunas Ramanavicius [1] and Almira Ramanaviciene [1,2,*]

[1] NanoTechnas—Center of Nanotechnology and Materials Science, Institute of Chemistry, Faculty of Chemistry and Geosciences, Vilnius University, Naugarduko st. 24, LT-03225 Vilnius, Lithuania; laura.sakalauskiene@chgf.vu.lt (L.S.); anton.popov@chgf.vu.lt (A.P.); asta.kausaite@chf.vu.lt (A.K.-M.); arunas.ramanavicius@chf.vu.lt (A.R.)

[2] Department of Immunology, State Research Institute Centre for Innovative Medicine, Santariskiu st. 5, LT-08406 Vilnius, Lithuania

* Correspondence: almira.ramanaviciene@chf.vu.lt

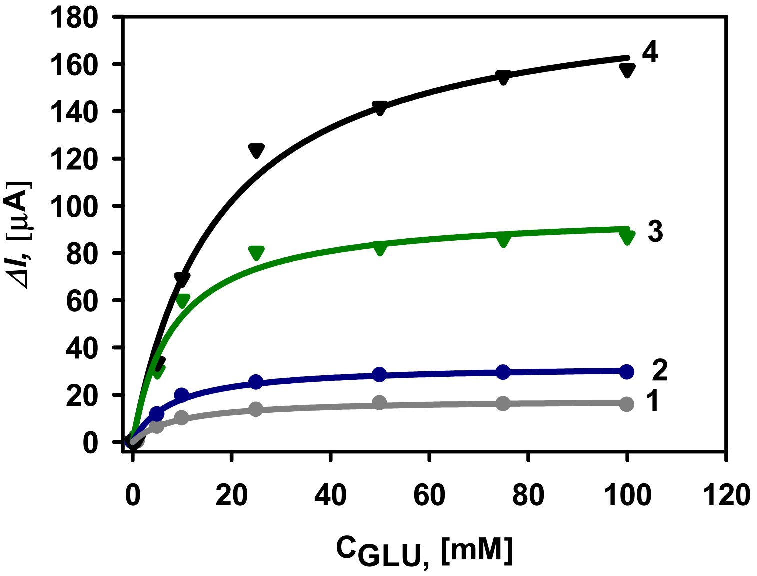

Figure S1. Calibration plots of glucose biosensors based on GA-GOx-SAM/DGNs/GR electrode without additional cross-linking with 25% glutaraldehyde vapour (curve 1), and after cross-linking for 3 min (curve 2), 10 min (curve 3) and 15 min (curve 4). The amperometric response at +0.3 V was registered in 0.05 M SA buffer with 0.1 M KCl (pH 6.0) in the presence of 2.0 mM PMS.

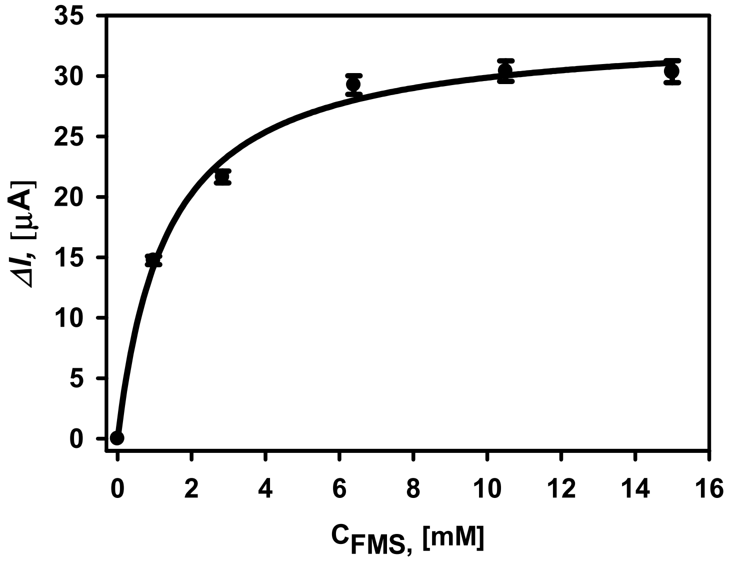

Figure S2. Selection of the optimal concentration of redox mediator PMS. Experiments were performed using GA-GOx-SAM/DGNs/GR electrode. The amperometric response at +0.3 V was registered in 0.05 M SA buffer with 0.1 M KCl (pH 6.0).

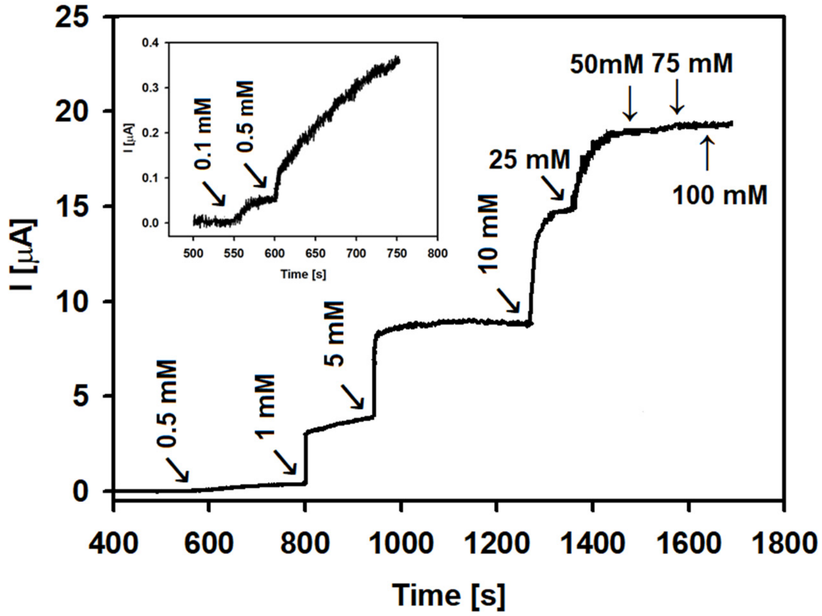

Figure S3. The amperogram of biosensor based on GOx/SAM/DGNs/GR electrode after addition of glucose. The current response was registered at +0.3 V in 0.05 M SA buffer with 0.1 M KCl (pH 6.0) in the presence of 6.0 mM PMS.

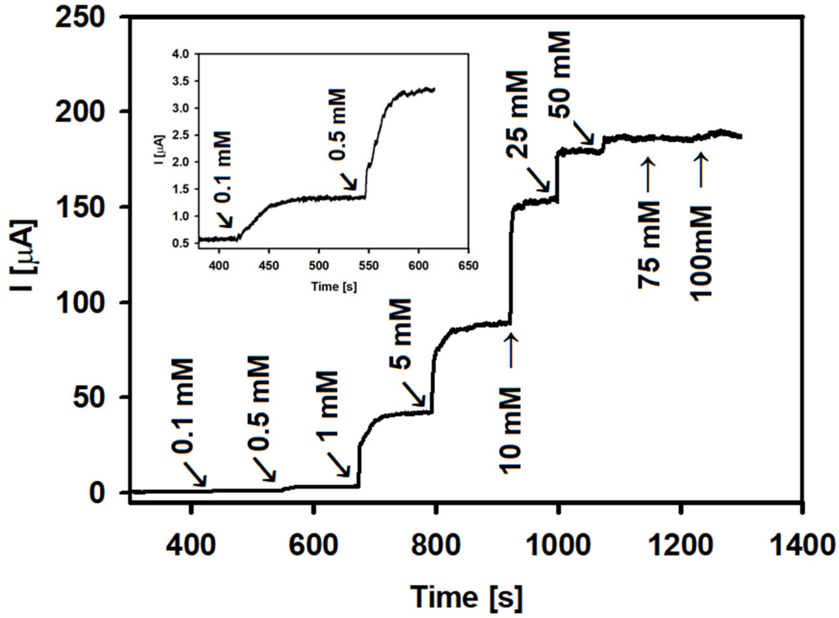

Figure S4. The amperogram of biosensor based on GA-GOx/DGNs/GR electrode after addition of glucose. The current response was registered at +0.3 V in 0.05 M SA buffer with 0.1 M KCl (pH 6.0) in the presence of 6.0 mM PMS.

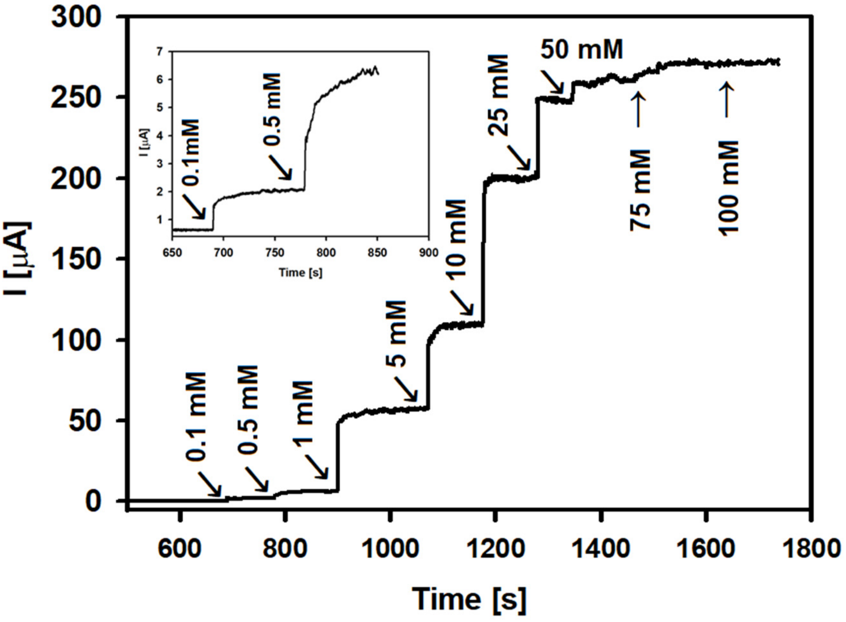

Figure S5. The amperogram of biosensor based on GA-GOx/SAM/DGNs/GR electrode after addition of glucose. The current response was registered at +0.3 V in 0.05 M SA buffer with 0.1 M KCl (pH 6.0) in the presence of 6.0 mM PMS.
